# The impact of camouflaging autistic traits on psychological and physiological stress: a co-twin control study

**DOI:** 10.1186/s13229-025-00695-9

**Published:** 2025-11-26

**Authors:** Sabela Conde-Pumpido Zubizarreta, Johan Isaksson, Åshild Faresjö, Tomas Faresjö, Angel Carracedo, Montse Fernández Prieto, Sven Bölte, Karl Lundin Remnélius

**Affiliations:** 1 Genomics and Bioinformatics Group, Center for Research in Molecular Medicine and Chronic Diseases (CiMUS), Universidade de Santiago de Compostela (USC), Santiago de Compostela, Spain; 2 Center of Neurodevelopmental Disorders (KIND), Department of Women’s and Children’s Health, Center for Psychiatry Research, Karolinska Institutet & Region Stockholm, Stockholm, Sweden; 3 Child and Adolescent Psychiatry Unit, Department of Medical Sciences, Uppsala University, Uppsala, Sweden; 4 Department of Health, Medicine and Caring Sciences, Division of Society and Health, Linköping University, Linköping, Sweden; 5 Genetics Group, Instituto de Investigación Sanitaria de Santiago (IDIS), Santiago de Compostela, Spain; 6 Centro de Investigación Biomédica en Red de Enfermedades Raras, Instituto de Salud Carlos III, Madrid, Spain; 7 Fundación Pública Galega de Medicina Xenómica, Servicio Galego de Saúde (SERGAS), Santiago de Compostela, Spain; 8 Fundación Pública Galega Instituto de Investigación Sanitaria de Santiago de Compostela (FIDIS), Santiago de Compostela, Spain; 9 Child and Adolescent Psychiatry, Region Stockholm, Stockholm, Sweden; 10 Curtin Autism Research Group, Curtin School of Allied Health, Curtin University, Perth, WA Australia

**Keywords:** Camouflaging, Masking, Stress, Cortisol, Hair cortisol concentration, Autism

## Abstract

**Background:**

Camouflaging autistic traits is suggested to increase stress and the risk of autistic burnout. However, the relationship with psychological and biological markers of stress and the influence of familial factors on this relationship remain unclear.

**Methods:**

In a neurodiverse twin sample (*N* = 315; 69 diagnosed with autism), we examined associations between camouflaging behaviors (operationalized as the discrepancy between the Autism Diagnostic Observation Schedule 2nd edition [ADOS-2] and the Autism Quotient [AQ]), rated stress-related symptoms, and biological long-term stress measured via hair cortisol concentration (HCC). Associations were analyzed across the full sample, accounting for age, sex, and HCC confounders, as well as within-twin pairs, implicitly adjusting for genetic and environmental confounding.

**Results:**

Across individuals, camouflaging was associated with increased HCC, particularly in autistic and adult subsamples, while associations with stress-related symptoms were only evident in adults. Within-pair analyses revealed no associations in the full sample, suggesting familial confounding. Interestingly, in adjusted within-pair models in autistic and adult twin-pairs, camouflaging was linked to *lower* HCC.

**Limitations:**

Only the adult participants provided self-reports of autistic traits (AQ) and stress-related symptoms, while parent-reports were used for the younger participants. This difference in reporting sources may have reduced the accuracy of data for the younger subgroups in our sample.

**Conclusions:**

The findings indicate that camouflaging is associated with increased biological long-term stress at the population level but that familial factors may influence this relationship. Future research is needed to explore these complex dynamics and their implications for mental health and adaptive functioning in autistic individuals.

**Supplementary Information:**

The online version contains supplementary material available at 10.1186/s13229-025-00695-9.

## Background

Camouflaging (or masking) is defined as the repertoire of verbal and non-verbal strategies employed by neurodivergent/autistic individuals to mitigate the visibility of their differences, serving an adaptive purpose of facilitating social interactions and inclusion [1]. While currently mainly studied in autism, camouflaging has been suggested to occur across neurodivergence, e.g., also in Attention-Deficit/Hyperactivity Disorder (ADHD) and developmental language disorder [2, 3]. The use of camouflaging strategies may be a compelled form of *impression management*, i.e., the ubiquitous human tendency to convey favorable impressions of oneself to others [1]. In line with this, camouflaging is correlated with dimensional autistic traits among non-autistic and autistic people, suggesting that camouflaging may be a strategy to make autistic traits less visible also among people who do not have an autism diagnosis [4–6].

Despite its adaptive function, emerging evidence show that camouflaging is linked to mental health problems among autistic individuals, including symptoms of exhaustion, depression, and anxiety, interpreted as tentative negative effects of masking [7, 8]. Camouflaging has been associated with internalizing symptoms also in non-autistic individuals, even when controlling for autistic traits [9]. Our research group recently found a link between camouflaging and poorer quality of life, beyond the influence of autistic traits, ADHD, and familial confounding from genetics and shared environment [10]. The taxing nature of camouflaging is illustrated in qualitative studies where autistic adults portray a highly stressful process involving continuous self-monitoring and observation of others [11]. Consistently, camouflaging is associated with self-reported stress in autistic as well as non-autistic individuals [9, 12, 13]. Given that camouflaging is hypothesized to be a main driver of *autistic burnout*, experienced as long-term exhaustion, loss of skills, and reduced tolerance to sensory stimuli, calls have been made for investigations into the relationship between camouflaging, burnout, and long term physical and mental health effects [14, 15].

Stress involves psychological perceptions, i.e., recognition of a challenge or perceiving that demands exceed individual resources, which activates the body’s physiological responses to stress, primarily orchestrated by the brain [16]. Cortisol is one of the hormones responsible for the physiological stress response and is produced by a neuroendocrine system called the hypothalamic-pituitary-adrenal (HPA) axis [17]. An increase in cortisol levels under an acute stressor is a natural response that normalizes as the individual adapts to the situation [16]. However, persistent stressors can lead to a dysfunctional stress response (i.e., with chronic higher or lower cortisol secretion) and lead to symptoms of burnout, depression, anxiety, and reduced stress tolerance [18]. Consistently, internalizing disorders have been associated with both hyperactivity and hypoactivity of the HPA axis [19, 20].

While associations between camouflaging and perceived stress are replicated in both autistic and non-autistic groups [13], investigations into the potential biological outcomes of such strategies are currently lacking. Camouflaging has been proposed as a form of chronic stress for autistic adolescents, as it is a response to the feeling of mismatch between external demands and internal capacity [21]. Ilen et al. [22] hypothesized that camouflaging could be a source of increased cortisol levels in young autistic females. Both activation of the HPA axis and camouflaging can be adaptive responses when experienced in lower quantities over shorter periods, but harmful to mental health if extensive and prolonged. Even though camouflaging has not been addressed, there is previous research into physiological stress and neurodevelopmental conditions (NDCs), focusing on children and adolescents and using saliva cortisol concentrations. Those findings indicate lower cortisol levels in ADHD and no clear pattern in autism [23–26]. However, cortisol levels in saliva only cover a spot time interval, being influenced by daily fluctuations and other factors [20, 27, 28].

Given that cortisol is incorporated into hair as it grows, measuring hair cortisol concentrations (HCC) has recently been suggested as a more stable measure of chronic biological stress response (i.e., accumulated stress over time [29]). HCC shows positive associations with salivary cortisol measures and with presence of cardiovascular diseases and traditional cardiovascular risk factors [30]. Also, HCC is associated with stressors such as chronic pain, chronic conditions, major life stressors (e.g., serious illness, death of a close relative), and long-term unemployment, supporting the use of HCC as a biomarker of long-term stress [20, 31, 32]. Extensive camouflaging could be hypothesized as a chronic stressor, especially in autistic and other neurodivergent groups who may be compelled to camouflage consistently across contexts in everyday life. While a causal influence of camouflaging on stress has been hypothesized [33], alternative explanations including confounding scenarios must first be ruled out. Especially familial factors, including genetic and environmental influences shared by family members, may act as confounders on the link between camouflaging and stress. For example, research has demonstrated that familial environmental influences, such as lower educational level in parents, are associated with higher HCC levels in their children [34]. In addition, HCC is found to be highly heritable in adolescents and young adults [35].

In summary, while previous research has shown associations between camouflaging and self-reported outcomes including perceived stress in autistic and non-autistic populations, the relationship with biomarkers of stress has currently not been investigated. Further, previous research has not accounted sufficiently for familial factors, which may confound the association. The present study aims to investigate the unique links between camouflaging and stress-related symptoms and a biomarker of stress (HCC) in a neurodiverse sample of comprehensively phenotyped autistic and non-autistic twins. Using a co-twin control design allows adjustment for a range of potentially confounding factors, including common genetic and shared environmental influences. We hypothesized that camouflaging would be associated with increased perceived stress-related symptoms and with higher HCC. Further, we expected that the association between camouflaging and stress would remain significant within twin-pairs when adjusting for shared genetic and environmental influences, highlighting an association beyond familial confounding.

## Methods

### Participants

The sample comprised 315 participants (91 monozygotic (MZ) pairs, 66 dizygotic (DZ) pairs, including one trio of DZ triplets; 52.4% females). In the sample, 21.9% had an autism diagnosis (39.1% females, see Table 1 for sample details), 28.3% had ADHD, 6% had other NDCs, e.g., specific learning disorder or developmental language disorder), and 28.9% had other psychiatric conditions (e.g., depression or anxiety conditions). In total, 12 twin pairs were concordant for autism (9 MZ pairs), while 44 twin pairs and one trio of triplets were discordant for autism (19 MZ pairs). Informed consent was obtained from participating twins and/or their parents depending on age, following the Declaration of Helsinki.

Sociodemographic information included parental country of birth and monthly household income. Among the mothers who reported their country of birth, 91.6% reported Sweden, 5.2% another European country, and 3.2% a non-European country. Regarding the fathers, 90.3% reported being born in Sweden, 5.8% in another European country, and 3.9% a non-European country. Information on parents’ country of birth was missing for two pairs. Monthly household income in Swedish crowns (10 Swedish crowns ≈ 0.87 Euro [€]) was reported by 97.5% of the participants’ parents: 3.9% of the participants had an income below 20,000, 25.1% between 20,000 and 40,000, 37.8% between 40,000 and 60,000, 21.2% between 60,000 and 80,000 and 12.1% above 80,000.

**Table 1:** Sample characteristics

	Full Sample						Autistic Subsample		Adult Subsample *	
	All		Females		Males					
	*N*	M (SD)	*N*	M (SD)	*N*	M (SD)	*N*	M (SD)	*N*	M (SD)
Age / range	315	16.76 (6.65) 8–38	165	18.81 (7.39) 8–38	150	14.51 (4.84) 8–36	69	14.93 (5.25) 8–28	148	22.35 (5.59) 15–38
CF_AQ/_ range	315	0 (0.23) -0.73-0.63	165	0.03 (0.21) -0.59-0.63	150	-0.04 (0.25) -0.73-0.45	69	-0.18 (0.29) -0.73-0.39	148	0.06 (0.21) -0.67- 0.63
ADOS CSS	315	2.61 (2.27)	165	2.07 (1.86)	150	3.2 (2.52)	69	5.64 (2.26)	148	1.98 (1.97)
AQ Total	315	17.90 (9.30)	165	16.10 (9.19)	150	19.88 (9.04)	69	25.26 (7.73)	148	14.55 (8.80)
SPS / range	310	4.29 (2.86) 0–13	161	4.57 (3.09) 0–13	149	4 (2.58) 0–12	67	5.08 (2.42) 0–11	145	4.15 (2.89) 0–13

	N	Median (IQR)	N	Median (IQR)	N	Median (IQR)	N	Median (IQR)	N	Median (IQR)
HCC / range	315	25.87 (32.34) 4.55-4306.33	165	29.31 (39.40) 4.55-1767.12	150	23.76 (26.53) 5.26-4306.33	69	25.87 (25.96) 5.26-378.15	148	25.44 (34.38) 5.26- 1079.72

*Note. SPS =* Stress Problems Score, *CF*_*AQ*_*=* Camouflaging Discrepancy Score, *ADOS CSS =* Autism Diagnostic Observation Schedule, Calibrated Severity Scores, *AQ* = Autism Quotient; *IQR =* Interquartile Range; *HCC =* raw Hair Cortisol Concentration values. * The adult subsample includes participants aged 15 to 38 who provided self-reports of both autistic traits and stress-related symptoms

### Procedure

The study used data from a twin sample enriched for NDCs, the Roots of Autism and ADHD Twin Study in Sweden (RATSS) [36, 37]. Participants in RATSS were mainly recruited from the population-based Child and Adolescent Twin Study in Sweden (CATSS) [38]. The recruitment in RATSS prioritizes twin-pairs where at least one twin scores high on the autism or ADHD domain of the “Autism-Tics, ADHD and other Comorbidities inventory” (A-TAC) [39] but also includes typically developing pairs with no evidence of NDCs. In RATSS, all families participated in comprehensive neurodevelopmental and psychiatric assessments during a 2½ day visit at a clinical research center. Eligibility criteria for the present study required both twins to have sufficient hair samples to obtain HCC values, data from the Autism Diagnostic Observation Schedule 2nd edition (ADOS-2) modules 3 or 4 [40–42], Autism Quotient (AQ) [43–45], and medical history information. From the original sample of 466 assessed individuals in RATSS, 72 individuals were excluded due to missing or insufficient hair samples. Participants without ADOS-2 module 3 or 4 assessments (*n* = 21), completely or partially (>5 items) missing AQ data (*n* = 8), or missing medical information (*n* = 2) were also excluded. Furthermore, 48 individuals were excluded for the following reasons: the twin or their co-twin had a diagnosis of intellectual disability (ID; *n* = 26), missing zygosity information (*n* = 6), or co-twins of different sex (*n* = 7). Finally, one twin pair from a family where two sets of twins participated in RATSS was excluded. Participants with an ID diagnosis were excluded because AQ was designed to measure autistic traits in individuals with average Intelligence Quotient (IQ) or above. After exclusions, the final sample encompassed 315 participants.

#### Diagnostic assessment

All diagnoses were clinical best estimate diagnoses according to the Diagnostic Statistical Manual of Mental Disorders – fifth edition (DSM-5) [46] made by a group of experienced clinicians. For autism, the assessment was supported by Swedish versions of the Autism Diagnostic Interview–Revised (ADI-R) [47] and the ADOS-2. The Kiddie Schedule for Affective Disorders and Schizophrenia (K-SADS) [48] was employed for assessing psychiatric disorders and ADHD in children, while the Diagnostic Interview for ADHD in adults (DIVA) [49] and the Structured Clinical Interview for DSM-IV (SCID) [50] were used for adults. Diagnosis of ID was supported by the Wechsler Intelligence Scale for Children - Fourth Edition (WISC-IV) [51] and the Wechsler Adult Intelligence Scale - Fourth Edition (WAIS-IV) [52], together with the Adaptive Behavior Assessment System–Second Edition (ABAS-II) [53]. Zygosity was determined on a panel of 48 single nucleotide polymorphisms [54]. In cases where DNA analysis was incomplete or of poor quality (10 pairs), a 4-item zygosity questionnaire was used.

### Measures

#### Camouflaging Autistic Traits

Camouflaging was operationalized as the discrepancy between clinician-rated autism characteristics (ADOS) and self-/parent-rated autistic traits (AQ), following previous camouflaging studies [33, 55].

The ADOS-2 is a standardized and semi-structured assessment of autistic characteristics, including communication, social interaction, and restricted and repetitive behaviors [40–42, 56]. The ADOS-2 Calibrated Severity Scores (CSS) was used as a measurement of ‘external’ behavioural characteristics related to autism that is comparable across ADOS-2 modules, ranging from 1 to 10.

The AQ is a 50-item questionnaire designed for self-reports of autistic traits in both autistic and non-autistic populations with average or above-average IQ [43−45]. In this study, the self-report version of the AQ was used whenever possible (*n* = 148). For participants where the self-report questionnaire was not available, the parent-reported adolescent (*n* = 89) and child version (*n* = 78) were employed. The binary scoring system for AQ responses was applied for all versions [57], yielding a maximum score of 50. Across versions, the AQ has demonstrated excellent test-retest reliability, and moderate to high internal consistency [43–45].

For the camouflaging discrepancy score, the ADOS CSS and the AQ were first standardized by mean-centring and divided by the maximum score. The AQ was standardized within each age-version. The discrepancy score (henceforth CF_AQ_) was calculated by subtracting the standardized ADOS CSS score from the AQ standardized score. Due to mean centering and scaling, CF_AQ_ ranges from − 1 to 1, where higher scores indicate increased camouflaging.

The Camouflaging Autistic Traits Questionnaire Swedish Version (CAT-Q/SE) was used to assess the convergent validity of the discrepancy measure of camouflaging [4, 5]. The CAT-Q is a self-report measure consisting of 25 items designed to quantify camouflaging strategies in autistic and non-autistic individuals. The Swedish validation study found that the CAT-Q/SE demonstrated good-to-excellent reliability, as well as support for the construct validity of the scale. A CAT-Q/SE total score was available for 160 individuals. For most participants, the CAT-Q/SE was collected after their visit to RATSS, with an average delay of 4.6 years.

#### Stress-related symptoms

The Stress Problems Scale (SPS) [58] from the Achenbach scales is a 7-items measure of stress-related symptoms derived from research on Post-Traumatic Stress Disorder (PTSD) using the Child Behavior Checklist for Ages 1½–5 (CBCL 1½–5) [59]. Originally, the scale was designed to capture PTSD symptoms or stress responses in children exposed to traumatic events, selecting 7 items that effectively discriminate between children who have versus have not experienced severe stress. The SPS ranges from 0 to 14. Psychometric evaluations of the SPS in preschool-aged children supports its reliability regarding test-retest stability [58]. All seven items included in the SPS in the CBCL 1½–5 have corresponding items on the CBCL 6–18, the Youth Self Report (YSR) [60], and the Adult Self Report (ASR) [61], which were used in the current study. Scores on these items from CBCL (*n* = 42 participants), YSR (*n* = 167), and ASR (*n* = 79) were aggregated to generate the SPS in our study.

#### Biological stress

Hair samples from the twins were collected during their visit to RATSS by a research nurse using sanitized scissors to cut approximately 10 to 20 strands of hair. Hair was cut from the scalp close to the skin by trained staff, as recommended by the Society of Hair Testing (SoHT) [62]. The hair was subsequently stored dry at room temperature in dark. Following Yang et al. [63] and Brænden et al. [64] we used competitive radioimmunoassay (RIA) to extract and analyse cortisol levels in hair. The analysis of cortisol levels is described in detail in the Supplementary Material.

#### Medical history

Information on previously reported confounders on HCC, i.e., chronic conditions, oral glucocorticoid intake, hydrocortisone cream/ointment use, and BMI [65–67], were obtained from assessments in RATSS including a parent-reported medical questionnaire designed to cover pre-, peri- and postnatal factors and child medical history, which has been validated with medical registry data [68]. 

### Statistical analyses

Analyses were performed in R [69] and in SPSS, version 28. For all analyses, a p-value < 0.05 was considered significant. This threshold was chosen because the co-twin control design, although allowing for stringent control of confounding factors, relies on intrapair differences, which inherently limits statistical power. To reduce the risk of Type II errors, a more conservative threshold was not applied.

Missing questionnaire items were imputed using the means of the available items. For the Achenbach scales (ASR, YSR, CBCL), imputation was used only when participants had a maximum of one missing item from the SPS (*n* = 4). Following Ashwood et al. [70], participants with AQ with less than 5 missings were imputed (*n* = 26). The CAT-Q/SE score from one participant with 1 missing item was imputed.

HCC was transformed using a natural logarithm, following previous literature [66]. HCC distribution [Figure S1] and transformation analysis are detailed in the Supplementary Material. The potential influence from chronic conditions, medication, and Body Mass Index (BMI) on HCC was tested using Wilcoxon rank-sum and Kruskal-Wallis tests (see Supplementary Material 1 for details). Only allergies and asthma showed significant associations with HCC levels. Based on this, asthma and allergies were included as covariates in the main analyses, in addition to the planned covariates sex and age. The mean strand length was 4.99 cm (SD = 2.32 cm), and the mean weight was 6.14 mg (SD = 1.02). Our measure of biological long-term stress is thus reflecting the cumulative effect of stress in cortisol levels for approximately five months.

Spearman correlations between the discrepancy operationalization of camouflaging and the CAT-Q/SE total score were carried out to examine the construct validity of the discrepancy score. Also, Spearman correlations between stress-related symptoms and HCC were assessed. The relationship between camouflaging and stress was assessed using regression models within the Generalized Estimating Equations (GEE) framework with doubly robust standard errors (drgee package in R), which does not make any distributional assumptions [71]. This approach allows the application of regression models across individuals in a twin sample with standard errors that account for the clustering in the data (i.e., between the two twins in a pair). First, a crude model was fitted in the full sample. Second, two adjusted models were fitted, the first controlling for sex and age, and the second also for asthma and allergy. This approach was used to examine the relationship between (a) camouflaging and stress-related symptoms, and (b) camouflaging and HCC.

To explore if the association between camouflaging and stress remained beyond genetic and shared environmental confounding, conditional linear GEE regression models were fitted. These models adjust for all factors shared within twin pairs, including shared environment (e.g., socioeconomic background, parental styles, parent age) and genetic factors which are shared completely by MZ twins and are ~ 50% shared by DZ twins [72]. The within-pair models also implicitly adjust for sex and age given that these factors are shared in all included twin-pairs. First, crude models, not split by zygosity, were fitted separately for (a) stress-related symptoms and (b) HCC. Second, allergy and asthma were added as covariates to the model. Third, zygosity was added as a factor, and both a crude model and an adjusted model were carried out. The within-pair analyses assess whether the twin who has a higher camouflaging discrepancy score in a twin pair shows increased or decreased stress compared to their co-twin. Twins that are discordant on exposure, covariates, and/or outcome are informative for the within-pair analyses. In the current sample, 89 MZ twin pairs had a camouflaging score discordance greater than 0 points, while 53 MZ twin pairs had a camouflaging score discordance of at least 0.1 points. Of the DZ twin pairs, 58 had a discordance greater than 0 and 44 had a discordance of at least 0.1 points for camouflaging.

The analyses were conducted in the full sample, as well as in the subsamples of older adolescent/adult participants (henceforth, “adult subsample” for brevity) and autistic participants to investigate the association between camouflaging and stress in these groups. To avoid loss of statistical power, the within-pair analyses in the autistic and adult subsamples were not split by zygosity. The analyses in the 148 adult participants were conducted to assess the associations among participants who self-reported both autistic traits and perceived stress-related symptoms. In the autistic group, the within-pair analyses were carried out in the 12 autism concordant twin pairs.

## Results

### Correlations between operationalizations of camouflaging and stress

The camouflaging discrepancy score did not significantly correlate with the CAT-Q total score in the full sample (*r* = 0.04, *p* = 0.58), but positive correlations were found in the autistic (*r =* 0.41, *p =* 0.03) and adult subsamples (*r* = 0.27, *p* = 0.01). No association was found between stress-related symptoms and HCC in the full sample (*r*= -0.02, *p* = 0.68), nor in the autistic (*r* = 0.15 *p =* 0.23) or the adult (*r=* -0.02, *p =* 0.80) subsamples.

### Across‑individuals analyses

The across-individuals associations between camouflaging discrepancy score and stress are presented in Table 2. In the full sample, camouflaging was not associated with stress-related symptoms. Increasing age and male sex were associated with a decrease in stress symptoms in both adjusted models. In contrast, camouflaging was associated with higher HCC in the full sample (*b* = 0.47, 95% CI = 0.06 to 0.88, *p* = 0.03), and this association remained significant after controlling for sex, age, asthma, and allergy in the adjusted models (*b* = 0.41, 95% CI = 0.00 to 0.81, *p* < 0.05).

In the autistic subgroup, camouflaging was not associated with stress-related symptoms. However, camouflaging was associated with an increase in HCC in the crude model (*b =* 0.62, CI = 0.16 to 1.10, *p* = 0.01) and when adjusting for sex and age (*b =* 0.62, 95% CI = 0.09 to 1.15, *p* = 0.02). Yet, the association attenuated and was not significant when also adjusting for allergy and asthma. In the adult subsample, camouflaging was associated with stress-related symptoms in the crude model (*b =* 4.08, 95% CI = 1.05 to 7.11, *p* = 0.01), and this association remained after adjusting for sex, age, allergy, and asthma (*b =* 4.27, 95% CI = 1.22 to 7.33, *p* = 0.01). Additionally, camouflaging predicted an increase of HCC in both the crude and the adjusted models. Plots showing the associations between camouflaging and stress-related symptoms and HCC in the full sample and subsamples can be found in the Supplementary Material [Figure S2 and Figure S3].

**Table 2:** Across-individuals association between camouflaging and perceived and biological long-term stress

	Full Sample			Autistic Subsample			Adult Subsample		
	Crude Model	Adjusted Model 1	Adjusted Model 2	Crude Model	Adjusted Model 1	Adjusted Model 2	Crude Model	Adjusted Model 1	Adjusted Model 2
	b/SE/p	b/SE/p	b/SE/p	b/SE/p	b/SE/p	b/SE/p	b/SE/p	b/SE/p	b/SE/p
Stress-related symptoms *									
Camouflaging	1.31/0.80/0.10	1.63/0.83 /0.05	1.62/0.83 /0.05	0.30/1.04/0.77	0.16/1.07 /0.88	-0.19/1.01/0.85	**4.08/1.55/0.01**	**4.24/1.56** **/0.01**	**4.27/1.56** **/0.01**
Sex		**-0.88/0.39** **/0.03**	**-0.89/0.40/0.03**		-0.98/0.68 /0.15	-1.19/0.67 /0.07		-0.65/0.58/0.23	-0.68/0.57/0.23
Age		**-0.10/0.03** **/** **<0.001**	**-0.10/0.03/<0.001**		-0.04/0.05 /0.41	-0.05/0.05 /0.39		**-0.18/0.05/<0.001**	**-0.18/0.05/<0.001**
Allergy			0.04/0.34 /0.90			0.59/0.58 /0.31			-0.15/0.49/0.75
Asthma			0.10/0.40 /0.80			0.52/0.81 /0.52			-0.49/0.56/0.38
*HCC*									
Camouflaging	**0.47/0.21/0.03**	**0.40/0.20** **/0.05**	**0.41/0.21/0.05**	**0.63/0.24/0.01**	**0.62/0.27** **/0.02**	0.48/0.27 /0.08	**0.62/0.29/0.03**	**0.61/0.29** **/0.04**	**0.62/0.28** **/0.03**
Sex		-0.09/0.16 /0.56	-0.14/0.15/0.38		-0.14/0.21/0.51	-0.22/0.20/0.29		0.14/0.27 /0.59	0.14/0.25 /0.57
Age		0.01/0.01 /0.66	0.01/0.01 /0.57		-0.01/0.01/0.60	-0.01/0.01/0.50		0.03/0.02 /0.24	0.03/0.02 /0.15
Allergy			**0.34/0.13** **/0.01**			0.33/0.19 /0.08			**0.49/0.21** **/0.02**
Asthma			0.27/0.18 /0.14			-0.03/0.21/0.88			0.03/0.19 /0.88

*Note. b =* regression coefficient, *SE =* standard error, p *=* p-value. The reference level for Sex is “Female,” and for Allergy and Asthma, it is “0,” indicating no Allergy or Asthma. Significant results are highlighted in bold

* Ten twin pairs were excluded due to missing SPS total score

### Within‑pair analyses

Within-pair analyses in the full sample did not find associations between camouflaging and stress-related symptoms or HCC (see Table 3), and not within MZ and DZ twin pairs separately [Supplementary Material, Table S1 and Figure S4]. The within-pairs analyses between camouflaging and stress in the autistic and adult subsamples are detailed in Table 4. In the autism concordant twins, camouflaging was not associated with stress-related symptoms. The association between camouflaging and HCC was not found in the crude model, but a negative association was found in the adjusted model (*b*= -2.54, 95% CI= -3.50 to -1.57, *p* < 0.001). Also, allergy was associated with an increase in HCC, while asthma was associated with decrease in cortisol. In the adult subsample, within-pair analyses found that camouflaging was not associated with stress-related symptoms. The crude model yielded a trend-level non-significant negative association between camouflaging and HCC. However, when adjusting for other covariates, camouflaging showed a significant negative association with HCC (*b*= -0.66, 95% CI= -1.31 to -0.02, *p =* 0.04).

**Table 3:** Within-pair association between camouflaging and stress in the full sample

	Crude Model	Adjusted Model
	b/SE/*p*	b/SE/*p*
Stress-related symptoms*		
Camouflaging	0.12/1.19/ 0.92	0.07/1.21 /0.95
Allergy		-0.14/0.41 /0.72
Asthma		-0.16/0.63 /0.80
*HCC*		
Camouflaging	-0.22/0.23 /0.34	-0.24/0.23 /0.30
Allergy		**0.22/0.11 /0.04**
Asthma		-0.15/0.13 /0.25

*b =* regression coefficient, *SE =* standard error, *p =* p-value. Significant associations are indicated in bold

*Ten twin pairs were excluded due to missing SPS total score

**Table 4:** Within-pair association between camouflaging and stress in the autistic and adult subsamples

	Autistic Subsample		Adult Subsample	
	Crude Model	Adjusted Model	Crude Model	Adjusted Model
	b/SE/p	b/SE/p	b/SE/p	b/SE/p
Stress-related symptoms*				
Camouflaging	3.61/3.99/0.37	1.50/4.18/0.72	3.26/3.14/0.30	3.37/3.40/0.32
Allergy		0.05/1.31/0.97		-0.53/0.71/0.46
Asthma		-2.66/2.16/0.22		0.39/0.74/0.60
*HCC*				
Camouflaging	-0.15/1.35/0.91	**-2.54/0.49/<0.001**	**-**0.55/0.32/0.09	**-0.66/0.33/0.04**
Allergy		**2.04/0.50/<0.001**		0.13/0.19/0.51
Asthma		**-0.42/0.12/<0.001**		-0.25/0.18/0.17

*Note. b =* regression coefficient, *SE =* standard error, *p =* p-value, *HCC =* Hair Cortisol Concentration, *SPS =* Stress Problems Score. The reference level for Allergy and Asthma is “0,” indicating no Allergy or Asthma. Significant results are highlighted in bold

## Discussion

This is the first study to investigate the association of camouflaging behaviors with markers of both psychological and physiological stress. Additionally, we examined the impact of familial confounding from genetics and shared environment on the link between camouflaging and stress using a co-twin control design. The study only found an association between camouflaging and stress-related symptoms in adults. However, a positive association between camouflaging and long-term biological stress was found across the full sample, as well as in autistic and adult subsamples, suggesting increased levels of long-term cortisol in those who camouflage extensively. Contrary to our hypothesis, the relationship between camouflaging and HCC was lost in the within-pair analyses, suggesting potential familial confounding. Interestingly, the adjusted within-pair analyses in the autistic and adult twin-pairs suggested that increased camouflaging could be associated with a *decrease* in HCC when the influence from familial factors are adjusted for.

The current study did not find an association between camouflaging and stress-related symptoms in the full sample or among the autistic participants. However, a positive association in the adult subsample was found, which remained significant after controlling for covariates. Potentially, the difference between subsamples may be due to age differences, where lacking associations in the full sample and autistic subsample may reflect the inclusion of children in these groups, who may both camouflage to a lesser degree and experience less of a negative impact on stress-related symptoms [5, 73, 74]. The transition to adulthood presents significant challenges due to increasing demands on social skills, greater exposure, financial independence and autonomy and higher levels of responsibility [75]. These demands can lead to increased stress in non-autistic adults but are particularly burdensome for autistic young adults [76]. Additionally, Taylor et al. [77] found that when facing social evaluation, autistic adults seem to experience anticipatory stress more intensely and sustain stress for longer compared to autistic adolescents, potentially reflecting the role of camouflaging in exacerbating both subjective and physiological stress. The difference between subsamples may also reflect methodological differences, where only adults provided self-reports of both autistic traits included in the discrepancy camouflaging measure and of subjective stress. The parent-reports, which were included in both the full sample and the autistic subsample, may not capture the subjective experience of autistic traits and/or stress-related symptoms as accurately as first-hand reports. Nevertheless, the association between camouflaging and self-reported stress among the autistic and non-autistic adults in our sample are in line with previous findings [9, 12]. 

Camouflaging was positively associated with HCC in the full sample, as well as in the subsamples of autistic and adult individuals. This provides evidence that camouflaging behaviors are linked to increased physiological stress, possibly through prolonged activation of the HPA axis. While it has been proposed to be a main stressor for autistic individuals [15, 21], our study is the first to observe a link between camouflaging behaviors and a physiological measure of stress. Our across-individuals findings suggest that camouflaging may play a role in the heightened levels of cortisol in hair and saliva found in some studies comparing autistic children to their neurotypical peers [78]. Yet, among autistic participants the association was attenuated after accounting for asthma and allergy, suggesting that these chronic illnesses may be confounding the association. Among adults, camouflaging behaviors were linked to both stress-related symptoms and HCC, highlighting the compounded burden of camouflaging in adulthood. These findings suggest that camouflaging may become increasingly draining with age, as individuals invest more time and energy in performing masking strategies [73]. The long-term effects of camouflaging on mental health may be mediated by stress and exhaustion as suggested by research on autistic burnout and recently supported in a large sample of autistic adults [79]. Our results highlight that further study into the relationships between camouflaging, stress-related symptoms, and HPA activity is warranted for insights into risk factors of mental health problems among autistic people.

While the across-individuals analyses suggest that extensive camouflaging is associated with increased HCC, results from the within-pair analyses were mixed. In the full sample, the association was lost within twin pairs suggesting familial confounding. This may reflect shared environmental factors such as prenatal exposures, including gestational age and maternal stress, as well as postnatal influences like socioeconomic status (SES), where disadvantaged SES may influence both stress-related symptoms and cortisol levels [65]. In addition, HCC is heritable [35], and our findings may reflect genetic confounding on the link between camouflaging and cortisol levels. Future research should investigate genetic and environmental influences on camouflaging and on bivariate relationships with mental health outcomes including stress, using classic twin design.

Further, the fully adjusted within-pair analyses in the autistic and adult subgroups suggest that adjusting for familial factors resulted in a *negative* association between camouflaging and HCC. These findings need to be replicated given the relatively small number of participants in these subsamples. However, the results could imply that a negative association between these factors in adults and autistic people is obscured in across-individual analyses but emerges when familial factors are controlled for. One possibility is that autistic and adult twins who camouflage extensively and more “successfully” than their co-twins are less subjected to stigma and bullying from others, thereby decreasing the risk of long-term high levels of cortisol compared to their co-twin. Another possibility, highlighting the complexity of long-term stress which may result in both an up-regulated and a down-regulated HPA axis, is that a high degree of camouflaging over time contributes to long-term stress exposure which could lead to a suppression of the HPA axis and, therefore, to blunted cortisol levels. This interpretation is in line with previous research suggesting that exposure to long-term stress may result in reduced cortisol levels [66]. It is possible that camouflaging acts as a persistent stressor which contributes to a dysregulation of the HPA axis. This could mean that extensive camouflaging among autistic people and adults might initially elevate cortisol levels but later fatigues the HPA axis, leading to reduced cortisol responses. This hypocortisolism has been linked to stress-related conditions including chronic fatigue syndrome, post-traumatic stress disorder, and burnout [65], suggesting a potential pathway from camouflaging to specific mental health problems via down-regulation of the HPA axis. Speculatively, this could be a mechanism behind autistic burnout. Future research needs to apply longitudinal designs to better understand the association between camouflaging and stress.

### Limitations

The present study comes with some limitations. First, the use of an indirect, discrepancy-based measure of camouflaging behaviors could have an impact on the results validity. For younger individuals, we used the parent-report versions of the AQ as a proxy of “internal autistic traits”. It has been suggested that parent-reports on the AQ yield slightly lower scores than self-report [44], which could potentially affect the estimation of camouflaging scores in these groups. The discrepancy score was not associated with self-reported camouflaging on the CAT-Q/SE in the full sample, which may be due to the CAT-Q/SE data being collected later than the other variables. It could also reflect that our discrepancy score may have captured the camouflaging construct more accurately among the autistic and adult participants than in the full sample. Camouflaging strategies are not exclusive to autistic individuals, and previous studies have applied the discrepancy approach in studies that included non-autistic participants [6], and individuals with a wide range of autistic traits (e.g. [6, 33, 80]). Still, our sample was neurodiverse, including both participants diagnosed with autism and/or ADHD, and those without NDCs. The inclusion of young participants and twins with lower levels of autistic traits may have reduced the validity of the discrepancy measure in the full sample. Future research is necessary to further investigate the construct validity of camouflaging operationalizations in autistic and non-autistic populations.

Still, among autistic and adult subsamples, correlations between the camouflaging operationalizations were small-to-medium, in line with previous findings [6] suggesting that discrepancy scores and self-reported camouflaging captures overlapping but not identical phenomena. Potentially, discrepancy scores capture “successful” masking, resulting in autistic traits being less visible to an outside observer, whereas self-reports on the CAT-Q capture the individual’s intention to camouflage, whether “effective” or not. Our findings support previous studies showing that measures capturing camouflaging intent and measures capturing camouflaging efficacy are not redundant but rather complementary. Given the ongoing debate about the construct validity of the discrepancy method [81, 82], future replication of our findings using other camouflaging operationalizations are needed.

Second, our measure of stress-related symptoms is not an established measure of psychological stress, and it has not been validated in adolescent and adult populations. Also, the SPS does not fully capture important aspects of psychological stress, i.e., it does not pick up on perceptions of uncontrollability or that external demands exceed individual capacities to manage them. Further, we only had self-rated stress from the adult subgroup. This could have influenced our results and the lacking association between stress-related symptoms and HCC. Still, this is not an uncommon finding in previous research which has provided mixed support for the link between psychological and biological stress [20]. 

Third, the within-pair analyses depend on twin-pairs that are discordant on exposure or outcome, and thus the statistical power in these analyses are reduced compared to analyses across individuals. However, while the study was likely not adequately powered for small effects, the co-twin control design has been used previously in comparable samples to discover medium-to-large effects (e.g., Lundin Remnélius et al. [10]). Still, although the total sample was of reasonable size for a twin study, the subgroups of autistic and adult individuals were smaller. On the other hand, the twin sample was comprehensively phenotyped, including assessments of NDCs and cognitive testing by experienced clinicians, and a marker of biological stress. The co-twin control design allows adjustment for a range of confounding variables, yielding a degree of control over confounding variables which is rarely matched in camouflaging research.

Fourth, although the within-pair analyses provide stringent control for confounding, the current study could not account for all factors that might influence both camouflaging and stress. For example, while camouflaging in autism has been shown to at least partly mediate the link between stigma and mental health problems, stigma is also directly associated with mental health [83], and could be a contributing factor to stress among people with elevated autistic traits, beyond camouflaging. Additionally, we did not control for other psychiatric conditions such as anxiety and depression which could be associated with stress and may influence camouflaging behavior. However, given that internalizing symptoms have been linked to both increased and decreased cortisol levels and could potentially mediate the relationship between our main variables, adjusting for psychiatric conditions is not straightforward. Future research on this topic should address the influence of other psychiatric conditions.

Fifth, the co-twin control design does not rule out the possibility of reversed causation, or a potential bidirectional relationship between camouflaging and stress. In addition, due to the limited sample size, it was not possible to split the autistic and adult subsamples by zygosity, which could have yielded a clearer picture on potential genetic confounding. Finally, the results cannot be generalized to participants with an ID diagnosis.

## Conclusions

In conclusion, this study provides evidence for a link between camouflaging behaviors and the regulation of the HPA axis. A positive association between camouflaging behaviors and HCC was found, suggesting that individuals engaging in higher levels of camouflaging display increased cortisol levels. Among adults, camouflaging behaviors were linked to both stress-related symptoms and HCC, highlighting the compounded burden of camouflaging in adulthood. The within-pair analyses suggest potential familial confounding on the camouflaging-stress relationship, indicating that genetic and shared environmental factors may play a role. Interestingly, in adjusted models within concordant autistic and adult twin-pairs, the twin who camouflaged more had lower cortisol levels than their co-twin, suggesting the possibility that persistent camouflaging may result in a down-regulation of the HPA axis beyond familial factors. While camouflaging may partly act as a protective coping mechanism from negative impacts on mental health (e.g., bullying), it may also pose as a persistent stressor which exacerbates stress in social situations. Consistent use of taxing camouflaging strategies may initially increase HPA activity and subsequently lead to suppressed cortisol levels, increasing risk for exhaustion, autistic burnout, and other mental health problems. Further research should combine self-ratings on validated measures of perceived stress with biological markers in longitudinal designs, to advance our understanding of the tentative impact of camouflaging on stress suggested by our findings.

## Supplementary Information

Below is the link to the electronic supplementary material.

Supplementary Material 1

## Data Availability

The RATSS data analysed during the current study are not publicly available due to restrictions imposed by the ethics approvals but can be available from the corresponding author on reasonable request.

## Abbreviations

ADHD: Attention-deficit/hyperactivity disorder
ADOS: Autism diagnostic observation schedule
AQ: Autism quotient
ASR: Adult self report
CAT-Q: Camouflaging Autistic Traits Questionnaire
CATSS: Child and adolescent twin study in Sweden
CBCL: Child behavior checklist
CF_AQ_: Camouflaging discrepancy score
CSS: Calibrated severity scores
DSM-5: Diagnostic statistical manual of mental disorders – fifth edition
DZ: Dizygotic
GEE: Generalized estimating equations
HCC: Hair cortisol concentration
HPA: The hypothalamic-pituitary-adrenal
ID: Intellectual disability
IQ: Intelligence quotient
MZ: Monozygotic
NDC: Neurodevelopmental conditions
PTSD: Post-traumatic stress disorder
RATSS: Roots of autism and ADHD twin study in Sweden
SPS: The stress problems scale
YSR: Youth self report
