# Supplementary material for: The impact of camouflaging autistic traits on psychological and physiological stress: a co-twin control study
Source: Mol Autism. 2025 Nov 26;16:59. doi: 10.1186/s13229-025-00695-9 (PMC12659362; doi:10.1186/s13229-025-00695-9)
Supplement: Supplementary file 1 — Supplementary Material 1 [file 13229_2025_695_MOESM1_ESM.docx]

**Supplementary Material**

**Supplementary Material About Hair Sampling and Cortisol Concentration Analysis**

***Hair Sampling***

Following Yang et al.^1^ and Brænden et al.^2^ we used competitive radioimmunoassay (RIA) for extract and analyse cortisol levels in hair. The hair samples were cut into smaller pieces weighting 5 to 7 mg, frozen for 2 min in liquid nitrogen in a test tube, and grinded together with a 5 mm steel ball using a Retch Cryo Mill (20 HZ) for 2 min. A volume of 1 ml of methanol was added to each tube and then, the samples are incubated overnight on a moving platform. Then, 0.8 ml of the methanol supernatant is removed and lyophilized using a Savant Speed Vac Plus SC210A. The extracts of hair samples were dissolved in a radioimmunoassay buffer. Cortisol concentrations were analyzed as described by Morelius et al.^3^. The primary antibody used was a rabbit polyclonal antibody “Cortisol 3”, catalog number MBS535414 (MyBioSource, San Diego, USA). Its reactivity profile is as follows: cortisol 100%, prednisolone 37%, 11-deoxycortisol 5%, corticosterone 3%, and cortisone<1%. The radioligand (radiolabelled cortisol) competes for the same binding sites as cortisol on the antibody. The secondary solid-phase antibody was anti-rabbit Sac-Cel (AA-Sac1, ImmunoDiagnostic Systems Ltd, Boldon, England) and it is used to separate the free sections from the ones that are connected to the primary antibody. The amount of radiation measured is inversely proportional to the amount of cortisol in the sample. The extracted cortisol is analysed, ensuring that inter-assay variations are less than 8% and intra-assay variations less than 7%.^4^ Furthermore, the gamma radiation used for cortisol detection in the RIA-method is not influenced by coloured hair.^2,5^

***Hair Cortisol Concentration (HCC) Distribution and Transformation Analysis***

Raw hair cortisol concentrations (HCC, pg/mg) had an interquartile range (IQR) of 32.34 (Q1=15.48, Q3 = 47.81) with high skewness (skew = 11.29) and kurtosis (kurt = 154.49), thus requiring a natural logarithm transformation. Post-transformation, the interquartile range (IQR) of log-transformed HCC (henceforth HCC) was reduced to 1.13 (Q1 = 2.74, Q3 = 3.87, skew=1.39, kurt = 6.32).

The Shapiro-Wilk test confirmed non-normal distribution of HCC in both the full sample (*W* = 0.91, *p* < 0.001) and the adult subsample (*W* = 0.92, *p* < 0.001), while autistic participants showed a normal distribution (*W* = 0.97, *p* = 0.16). HCC distribution for the full sample and subsamples is represented in Figure S1.

**Figure S1.**

*HCC distribution in the full sample (a) and autistic (b) and adult subsamples.*


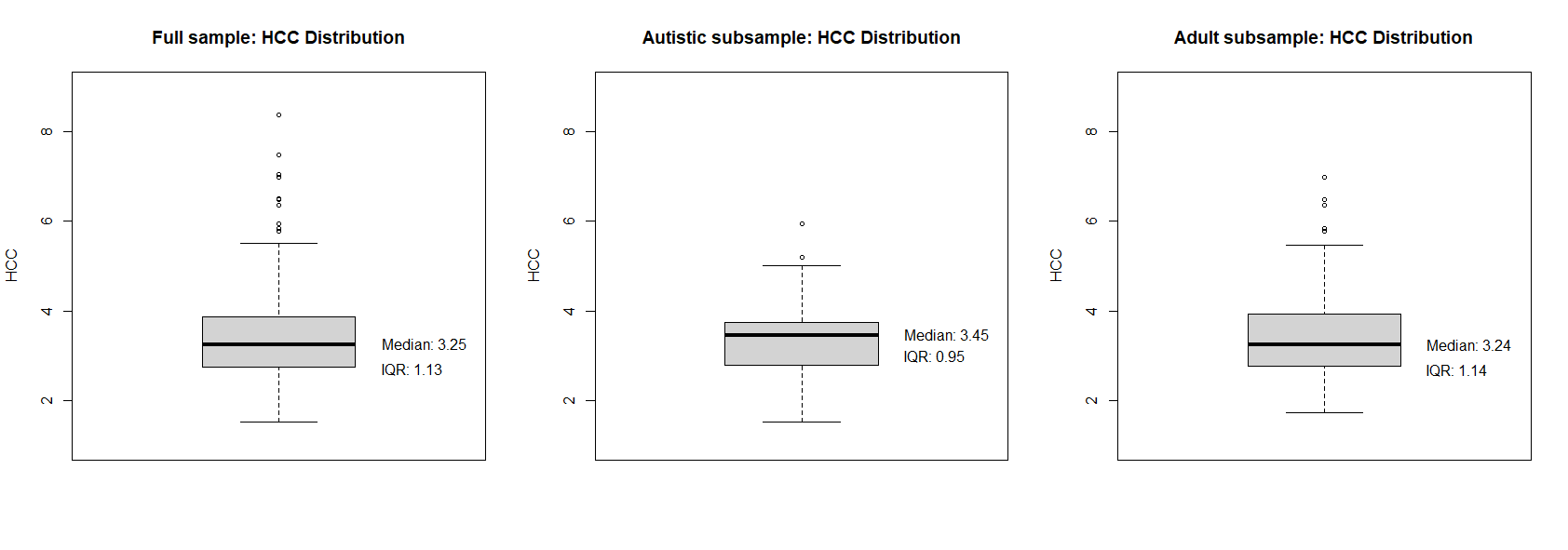


*Note. HCC =* Hair Cortisol Concentration, *IQR =* Intercuartile Range

**HCC Confounders Distribution and Associations**

The most reported chronic conditions included allergies (36.5%), eczema (26.0%) and asthma (19.7%). Additionally, 3.5% of the participants were taking steroid-based medication (e.g., inhaled budesonide, or mometasone nasal spray). Regarding BMI, 74.0% participants was classified as “normal weight”, 13.7% as “overweight”, 6.3% “underweight” and 6.0% were “obese”, using ISO-recommended cut-offs for children and adolescents under 19 years old.^6,7^ Participants with allergies exhibited higher HCC levels than those without allergies (*W* = 9379.5, *p* = 0.01) and the same was found for participants with asthma compared to those without (*W* = 6159, *p* = 0.01). No significant differences in HCC levels were found between different BMI classifications, steroid medication use, or other chronic conditions.

**Figure S2.**

*Association between camouflaging and stress-related symptoms in the full sample and autistic and adult subsamples.*


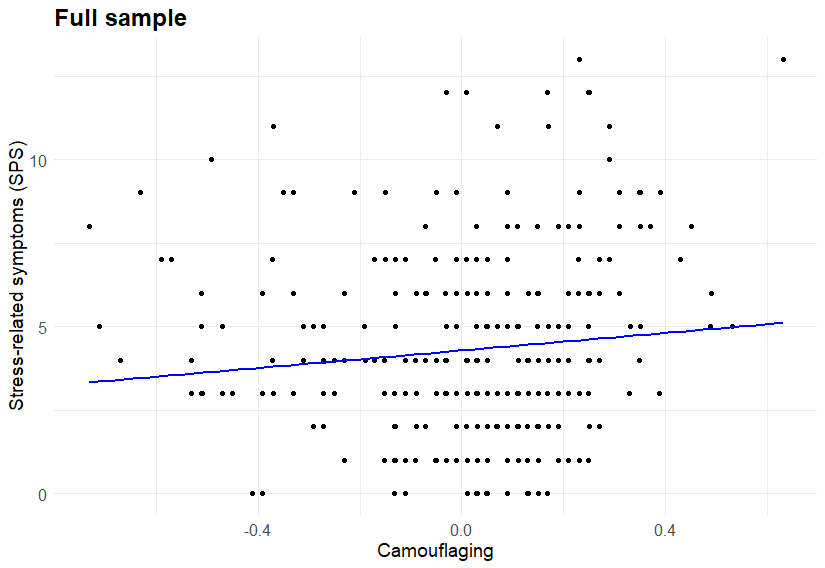


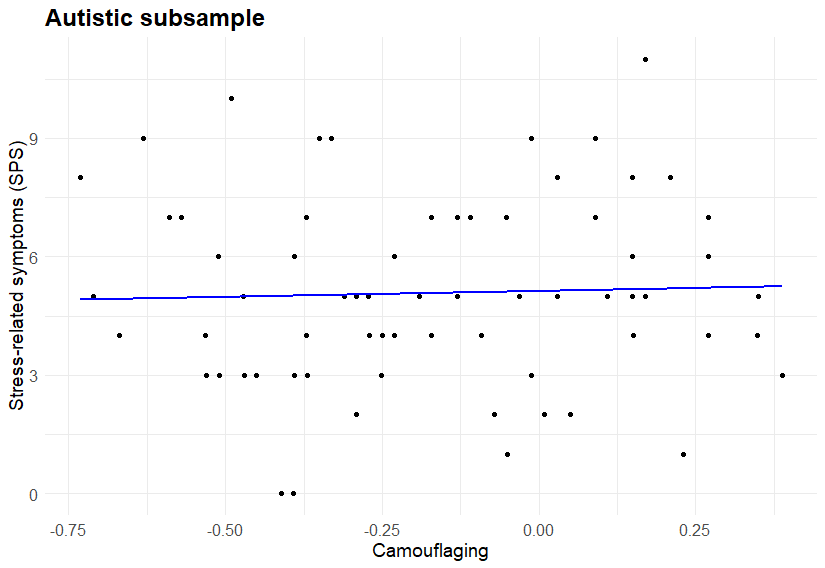

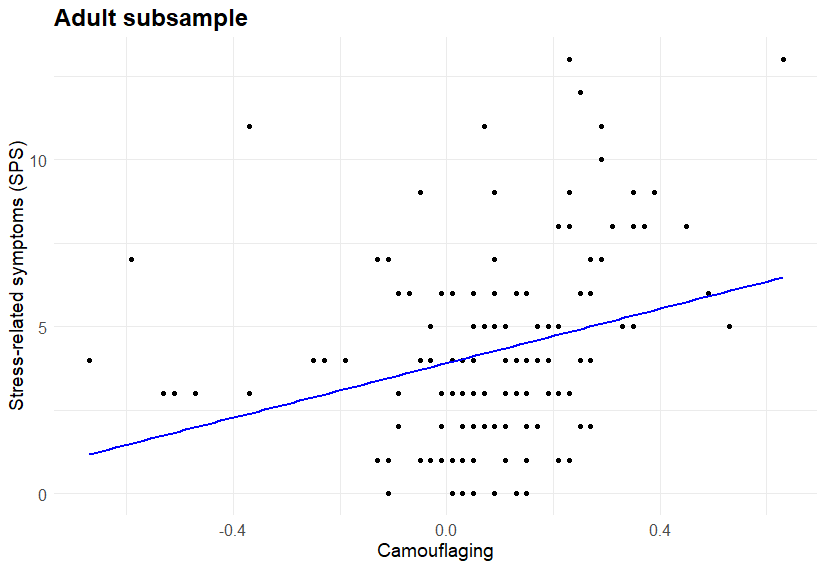


**Figure S3.**

*Association between camouflaging and HCC in the full sample and autistic and adult subsamples.*


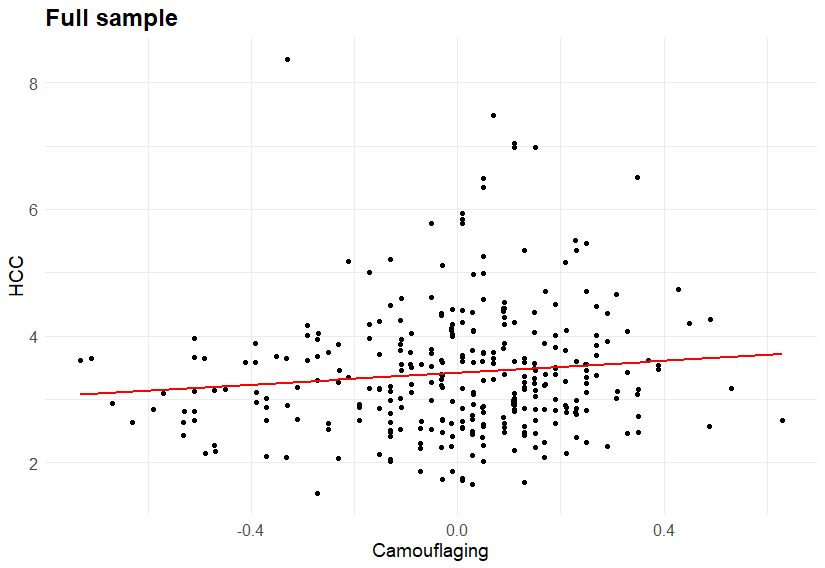

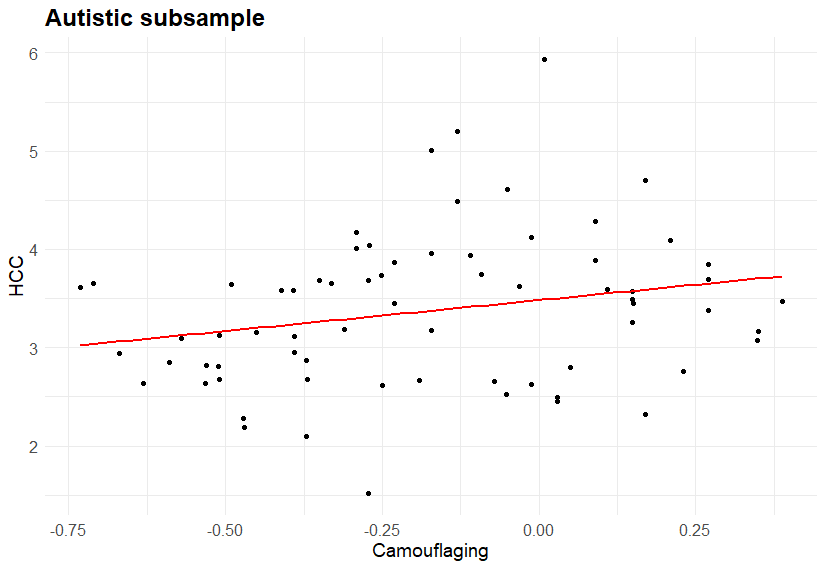


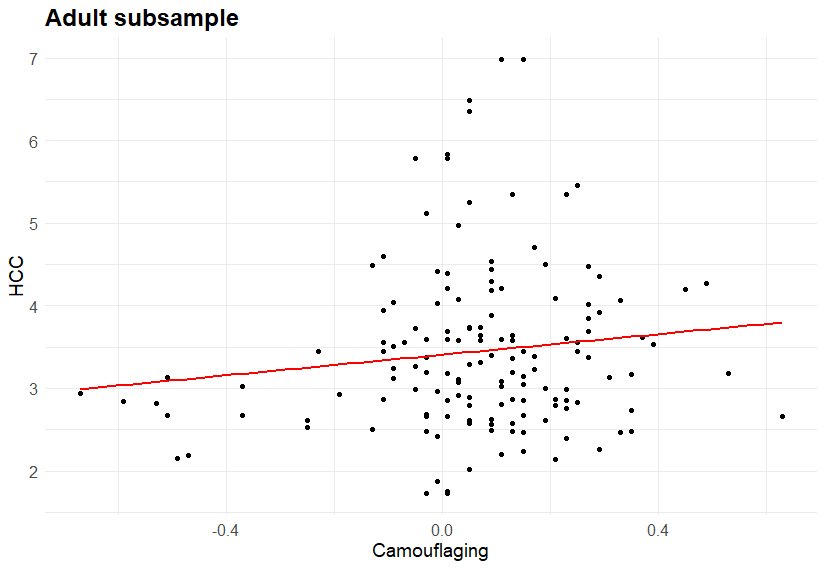


*Note. HCC =* Hair Cortisol Concentration

**Table S1**

*Within-pair association between camouflaging and stress in MZ and DZ twins.*

|  | MZ | | DZ | |
| --- | --- | --- | --- | --- |
|  | Crude Model | Adjusted Model | Crude Model | Adjusted Model |
|  | b/SE/p | b/SE/p | b/SE/p | b/SE/p |
| *Stress-related symptoms* |  |  |  |  |
| Camouflaging | -0.88/1.42/0.54 | -0.90/1.42/0.53 | 0.76/1.69/0.66 | 0.70/1.71/0.68 |
| *HCC* |  |  |  |  |
| Camouflaging | -0.33/0.28/0.24 | -0.36/0.29/0.23 | -0.14/0.33/0.66 | -0.16/0.33/0.63 |

*Note.* MZ = monozygotic twin pairs*,* DZ = dizygotic twin pairs*, b =* regression coefficient, *SE* = standard error, *p=* p-value. Significant associations are indicated in bold.

**Figure S4**

Within-pair associations between camouflaging and stress-related symptoms and HCC in MZ- and DZ-pairs. The twins in each pair are shown connected with a line.


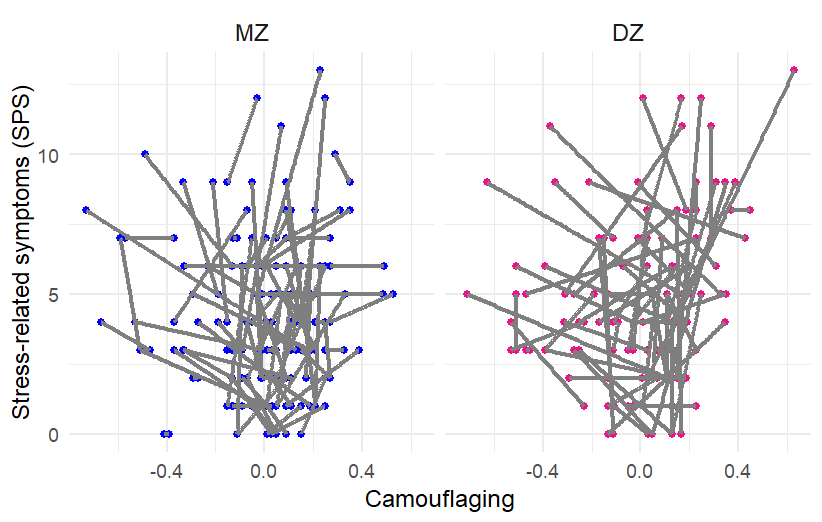

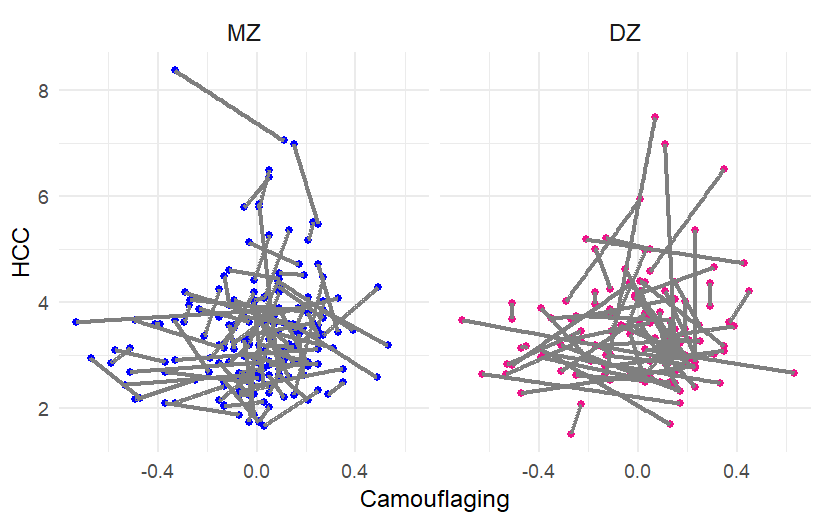


*Note. HCC =* Hair Cortisol Concentration

**References**

1. Yang HZ, Lan J, Meng YJ, Wan XJ, Han DW. A preliminary study of steroid reproductive hormones in human hair. J Steroid Biochem Mol Biol. 1998;67(5–6):447–50. doi:10.1016/s0960-0760(98)00120-4.
2. Brænden A, Lebena A, Faresjö Å, Theodorsson E, Coldevin M, Stubberud J, et al. Excessive hair cortisol concentration as an indicator of psychological disorders in children. Psychoneuroendocrinology. 2023;157:106363. doi:10.1016/j.psyneuen.2023.106363.
3. Morelius E, Nelson N, Theodorsson E. Salivary cortisol and administration of concentrated oral glucose in newborn infants: improved detection limit and smaller sample volumes without glucose interference. Scand J Clin Lab Invest. 2004;64(2):113–8. doi:10.1080/00365510410004452.
4. Karlén J, Ludvigsson J, Frostell A, Theodorsson E, Faresjö T. Cortisol in hair measured in young adults - a biomarker of major life stressors? BMC Clin Pathol. 2011;11:12. doi:10.1186/1472-6890-11-12.
5. Marteinsdottir I, Sydsjö G, Faresjö Å, Theodorsson E, Josefsson A. Parity-related variation in cortisol concentrations in hair during pregnancy. BJOG. 2021;128(4):637–44. doi:10.1111/1471-0528.16542.
6. Cole TJ, Bellizzi MC, Flegal KM, Dietz WH. Establishing a standard definition for child overweight and obesity worldwide: international survey. BMJ. 2000;320(7244):1240–3. doi:10.1136/bmj.320.7244.1240.
7. Cole TJ, Flegal KM, Nicholls D, Jackson AA. Body mass index cut offs to define thinness in children and adolescents: international survey. BMJ. 2007;335(7612):194. doi:10.1136/bmj.39238.399444.55.
